# Intravenous Thrombolysis for Pediatric Acute Ischemic Stroke

**DOI:** 10.1001/jamanetworkopen.2025.38191

**Published:** 2025-10-15

**Authors:** Peter B. Sporns, Kartik D. Bhatia, Prakash Muthusami, Carmen Parra-Farinas, Christine K. Fox, Adam A. Dmytriw, Sarah Lee, Jens Fiehler, Todd Abruzzo, Lisa Pabst, Stuart Fraser, Lisa R. Sun, Grégoire Boulouis, Tanja Burkard, Marios Psychogios, Thi Dan Linh Nguyen-Kim, Moritz Wildgruber

**Affiliations:** 1 Department of Radiology and Neuroradiology, Stadtspital Zürich, Zürich, Switzerland; 2 Department of Neuroradiology, University Hospital Basel, Basel, Switzerland; 3 Department of Diagnostic and Interventional Neuroradiology, University Medical Center Hamburg-Eppendorf, Hamburg, Germany; 4 Children’s Hospital at Westmead Clinical School, University of Sydney, Sydney, Australia; 5 Division of Paediatric Interventional Neuroradiology, Hospital for Sick Children, Toronto, Canada; 6 Department of Neurology and Department of Pediatrics, University of California San Francisco; 7 Neurovascular Centre, Divisions of Therapeutic Neuroradiology and Neurosurgery, St Michael’s Hospital, University of Toronto, Canada; 8 Division of Child Neurology, Department of Neurology, Stanford University, Stanford, California; 9 Department of Neurosurgery, Barrow Neurological Institute, Phoenix, Arizona; 10 Department of Radiology, Phoenix Children’s Hospital, Phoenix, Arizona; 11 Division of Pediatric Neurology, Department of Pediatrics, University of Utah School of Medicine, Salt Lake City; 12 Division of Child and Adolescent Neurology, Department of Pediatrics, The University of Texas McGovern Medical School, Houston; 13 Department of Neurology, Johns Hopkins School of Medicine, Baltimore, Maryland; 14 Diagnostic and Interventional Neuroradiology Department, CIC-IT 1415, CHRU de Tours, Tours, France; 15 Université de Tours, INSERM, Imaging Brain & Neuropsychiatry iBraiN U1253, 37032, Tours, France; 16 French Center for Pediatric Stroke, Paris, France; 17 Department of Radiology, LMU University Hospital, LMU Munich, Munich, Germany

## Abstract

This cohort study examines function and safety outcomes among children with pediatric acute ischemic stroke treated with intravenous tissue plasminogen activator compared with standard care.

## Introduction

Pediatric acute ischemic stroke (AIS) is associated with severe long-term sequelae.^1,2^ While intravenous (IV) thrombolysis is established in adults, its safety and efficacy in children remain uncertain.^3^ Current evidence derives from case series, and conducting randomized clinical trials in pediatric stroke is challenging due to regulatory and logistical barriers. The Thrombolysis in Pediatric Stroke trial was terminated early due to poor recruitment, but findings from Amlie-Lefond et al^4^ suggested that the risk of symptomatic intracerebral hemorrhage (SICH) in children treated with IV tissue plasminogen activator (TPA) may be comparable to adults.^4^ In this subanalysis of the Save ChildS Pro study, a large multinational pediatric AIS registry designed to assess the safety and outcomes of endovascular therapy (EVT) in children,^5^ we compare outcomes following IV-TPA vs best medical treatment (BMT) alone.

## Methods

This cohort study was conducted as part of the Save ChildS Pro study, which was approved by the ethics committee of the University of Münster and local ethics committees, in accordance with the Declaration of Helsinki, with waiver for informed consent because data collection was anonymized. The Save ChildS Pro cohort study included children aged 28 days to 18 years with AIS and intracranial arterial occlusion between January 1, 2020, and August 31, 2023.^5^ Patients who underwent EVT were excluded.

The primary outcome was the pediatric modified Rankin Scale (Ped-mRS) at 90 days. The primary safety outcome was the rate of SICH. Secondary outcomes included the Pediatric Stroke Outcome Measure (PSOM) at 90 days and changes in the Pediatric National Institute of Health Stroke Scale (Ped-NIHSS) from admission to discharge. Detailed methods are given in the eMethods in Supplement 1.

## Results

Of 208 patients (115 [55%] male; median [IQR] age, 9 [5-13] years), 90 were treated with IV-TPA (25 patients) or BMT alone (65 patients). Patients in the IV-TPA group were older than those in the BMT group (median [IQR] age, 10 [6-15] years vs 6 [2-10] years; *P* = .001). Symptom-onset to admission time was shorter for the IV-TPA group vs BMT group (median [IQR], 109 [56-217] minute vs 310 [210-480] minutes; *P* = .003). Baseline Ped-NIHSS and sex distribution were similar between groups (Table 1).

**Table 1.** Demographics, Clinical Presentation, and Stroke Etiology

Characteristic	Participants, No. (%)		*P* value
	IV-TPA (n = 25)	BMT (n = 65)	
Sex			
Female	11 (44)	27 (42)	.77
Male	14 (56)	38 (58)	
Age, y			
Mean (SD)	10.5 (5.4)	6.4 (5.0)	.001
Median (IQR)	10 (6-15)	6 (2-10)	NA
mRS prior stroke, median (IQR)	0 (0-0)	0 (0-1)	.02
Ped-NIHSS at admission			
Mean (SD)	11.6 (6.6)	9.5 (6.7)	.16
Median (IQR)	11 (8-13)	8 (4-12)	NA
Onset to admission, median (IQR), min	109 (56-217)	310 (210-480)	.003
Referral from other hospital	10 (40)	32 (49)	.36
Stroke etiology (CASCADE classification)			
Small vessel (1)	0	0	.05
Unilateral (2)	11 (44)	23 (35)	
Bilateral (3)	0	7 (11)	
Aorto-cervical (4)	2 (8)	3 (5)	
Cardioembolic (5)	3 (12)	12 (19)	
Other (6)	5 (20)	19 (29)	
Multifactorial (7)	4 (16)	1 (2)	

Abbreviations: BMT, best medical treatment; CASCADE, Childhood Arterial Ischemic Stroke Standardized Classification and Diagnostic Evaluation; IV-TPA, intravenous tissue plasminogen activator; mRS, modified Rankin scale; NA, not applicable; Ped-NIHSS, Pediatric National Institute of Health Stroke Scale score.

### Primary and Safety Outcomes

Patients treated with IV-TPA had lower Ped-mRS scores at 90 days vs the BMT group (median [IQR]. 1 [0-3] vs 2 [1-3]; *P* = .03), with lower scores associated with better functional outcomes (Table 2). Of 25 patients in the IV-TPA–only group, none experienced SICH; Of 20 patients treated with IV-TPA plus EVT, none had a SICH.

**Table 2.** Clinical and Safety Outcomes

Outcome	Participants, No. (%)		*P* value
	IV-TPA (n = 25)	BMT (n = 65)	
Ped-mRS at 90 d^a^			
Median (IQR)	1 (0-3)	2 (1-3)	.03
0	8 (32)	9 (14)	
1	7 (28)	14 (22)	
2	3 (12)	16 (25)	
3	6 (24)	10 (15)	
4	0	11 (17)	
5	1 (4)	1 (2)	
6	0	4 (6)	
PSOM at 90 d, median (IQR)	1 (1-2)	2 (1-3)	.005
Ped-NIHSS at discharge, median (IQR)	4 (1-9)	7 (3-13)	.06
Reduction in Ped-NIHSS, median (IQR)	−5 (−8 to −2)	−1 (−5 to 0)	<.001
Symptomatic ICH	0	1 (2)	NA

Abbreviations: BMT, best medical treatment; ICH, intracranial hemorrhage; IV-TPA, intravenous tissue plasminogen activator; NA, not applicable; Ped-mRS, Pediatric modified Rankin Scale score; Ped-NIHSS, Pediatric National Institute of Health Stroke Scale score; PSOM, Pediatric Stroke Outcome Measure.

^a^ Scores on the modified Rankin Scale range from 0 to 6; with higher scores indicating more severe disability.

### Secondary Outcomes

Patients in the IV-TPA group had lower PSOM scores at 90 days vs BMT (median [IQR], 1 [1 to 2] vs 2 [1-3]; *P* = .005). Improvement in Ped-NIHSS from admission to discharge was higher in the IV-TPA group (median [IQR] change, −5 [−8 to −2] vs −1 [−5 to 0]; *P* < .001).

## Discussion

In this cohort study of patients with AIS, use of IV-TPA was associated with lower median Ped-mRS and no increase in SICH. These findings align with the study by Amlie-Lefond et al,^4^ which estimated a 2.1% SICH risk in children treated with IV-TPA, similar to previous studies in young adults.

In our cohort, the older age of patients in the IV-TPA group may reflect hesitancy to treat younger children with thrombolysis. However, generalizability is probably not significantly impacted, as both groups were mostly school-aged children, with a greater proportion of preschool children in the BMT group.

This study has some limitations. The sample size was small, although this is the largest dataset on pediatric AIS comparing IV-TPA with a control group, to our knowledge. Additionally, we were unable to control confounding. Shorter onset-to-admission times in the IV-TPA group show bias by indication, which may partially account for better outcomes. This was a subanalysis of a study not originally designed to assess IV-TPA efficacy, which limits generalizability. Nonetheless, these findings suggest that safety concerns should not prevent treatment in children, especially when considering the potentially life-long disability and socioeconomical impact.^6^

## Notes

Supplement 1.
eMethods.

Supplement 2.
The SaveChildS Pro Investigators

Supplement 3.
Data Sharing Statement
