# Supplementary material for: Intravenous Thrombolysis for Pediatric Acute Ischemic Stroke
Source: JAMA Netw Open. 2025 Oct 15;8(10):e2538191. doi: 10.1001/jamanetworkopen.2025.38191 (PMC12529207; doi:10.1001/jamanetworkopen.2025.38191)
Supplement: Supplement 1. — eMethods. [file jamanetwopen-e2538191-s001.pdf]

Supplemental Online Content

Sporns PB, Bhatia KD, Muthusami P, et al; the SaveChildS Pro Investigators. Intravenous thrombolysis for pediatric acute ischemic stroke. *JAMA Netw Open*. 2025;8(10):e2538191. doi:10.1001/jamanetworkopen.2025.38191

eMethods.

This supplemental material has been provided by the authors to give readers additional information about their work.

© 2025 Sporns PB et al. *JAMA Network Open.*

eMethods

Study design and participants

Save ChildS Pro was an international prospective cohort study of children who presented with acute arterial ischemic stroke due to either large or medium vessel occlusion across 53 centres in Europe, North America, South America, Asia, and Australia (Sporns save childs Pro) and of whom 45 centres contributed eligible patient data that were included in the final analysis. Primary aim of the registry was to determine the difference between mRS scores before and 90 days after stroke in pediatric patients treated with either endovascular thrombectomy (EVT) and BMT only (including iv-tPA) .Registry inclusion criteria were patient age 28 days to 18 years, a clinical diagnosis of arterial ischemic stroke, and confirmed diagnosis of intracranial arterial occlusion consistent with symptoms including occlusion of terminal internal carotid artery, middle cerebral artery (M1, M2 segments), basilar artery, vertebral artery (V4 segment), anterior cerebral artery (A1, A2 segments), posterior cerebral artery (P1, P2 segments), and proximal superior cerebellar artery. Neonates with stroke were excluded. The protocol for the Save ChildS Pro study is available online.

Secondary data analysis for this study was undertaken between 1st November 2024 and 10th January 2025.

All children from the Save ChildS Pro study who underwent medical treatment were included and divided in two groups **1)** iv-tPA **2)** best medical treatment alone (BMT, no iv-tPA). Patients who had undergone endovascular thrombectomy (EVT) were not included in the analysis. The decision for iv-tPA versus BMT was left to the discretion of the treating pediatric stroke team, as no evidence-based standard operating procedures are established.

Primary and secondary outcomes

© 2025 Sporns PB et al. *JAMA Network Open.*

The primary clinical outcome was the Ped-mRS score at 90 days after stroke onset. The primary safety outcome was the rate of symptomatic intracranial haemorrhage (sICH), defined as intracranial extravascular blood associated with an increase in the Pediatric National Institute of Health Stroke Scale (PedNIHSS) score by 4 points or more.

Secondary outcomes included the Pediatric Stroke Outcome Measure (PSOM) 90 days following stroke onset and the decrease of the PedNIHSS from admission to discharge.

Statistical Analysis

Baseline characteristics and outcome data were compared between the iv-tPA and BMT groups. Continuous variables were compared using unpaired t-tests or Mann-Whitney U-tests, depending on data distribution. Categorical variables were compared using chi-squared tests or Fisher's exact tests. Ordinal data were analyzed using the Mann-Whitney U-test. Pearson correlation coefficients were used to determine treatment effects for main outcomes parameters (Ped-mRS and PSOM at 90 days). Statistical significance was set at $p < 0.05$. All statistical analyses were performed using SPSS (IBM Corp., Armonk, NY).

Data Availability

The data that support the findings of this study are available from the corresponding author upon reasonable request.

© 2025 Sporns PB et al. *JAMA Network Open.*

Save Childs Pro Investigators

Investigator	Affiliation
Peter B Sporns	Department of Neuroradiology, University Hospital Basel, Basel, Switzerland; Department of Diagnostic and Interventional Neuroradiology, University Medical Center Hamburg-Eppendorf, Hamburg, Germany; Department of Radiology and Neuroradiology, Stadtspital Zürich, Zürich, Switzerland
Kartik Bhatia	Department of Medical Imaging, Children's Hospital at Westmead, Sydney, NSW, Australia; Children's Hospital at Westmead Clinical School, Faculty of Medicine and Health, University of Sydney, Sydney, NSW, Australia
Todd Abruzzo	Department of Neurosurgery, Barrow Neurological Institute, Phoenix, AZ, USA; Department of Radiology, Phoenix Children's Hospital, Phoenix, AZ, USA
Lisa Pabst	Division of Pediatric Neurology, Department of Pediatrics, University of Utah School of Medicine, Salt Lake City, UT, USA
Stuart Fraser	Division of Child and Adolescent Neurology, Department of Pediatrics, The University of Texas McGovern Medical School, Houston, TX, USA
Melissa G Chung	Division of Critical Care Medicine and Division of Pediatric Neurology, Department of Pediatrics, Nationwide Children's Hospital and The Ohio State University, Columbus, OH, USA
Warren Lo	Department of Pediatrics and Department of Neurology, Nationwide Children's Hospital

© 2025 Sporns PB et al. *JAMA Network Open*.

	and The Ohio State University, Columbus, OH, USA
Ahmed Othman	Department of Neuroradiology, University Medical Center of the Johannes Gutenberg University, Mainz, Germany
Sebastian Steinmetz	Department of Neuroradiology, University Medical Center of the Johannes Gutenberg University, Mainz, Germany
Ulf Jensen-Kondering	Department of Radiology and Neuroradiology, University Medical Center Schleswig-Holstein, Kiel, Germany; Department of Neuroradiology, University Medical Center Schleswig-Holstein, Lübeck, Germany
Stefan Schob	Department of Radiology and Neuroradiology, University Hospital Halle, Halle, Germany
Daniel P O Kaiser	Institute of Neuroradiology, Medical Faculty and University Hospital Carl Gustav Carus, Dresden University of Technology, Dresden, Germany
Wolfgang Marik	Department of Neuroradiology, Medical University of Vienna, Vienna, Austria
Christina Wendl	Institute of Radiology, University Hospital Regensburg, Regensburg, Germany
Ilka Kleffner	Department of Neurology, University Hospital Knappschaftskrankenhaus, Ruhr University Bochum, Bochum, Germany
Hans Henkes	Neuroradiological Clinic, Katharinenhospital, Klinikum Stuttgart, Stuttgart, Germany
Hermann Kraehling	Clinic for Radiology, Department for Interventional Neuroradiology, University of Münster, Münster, Germany

© 2025 Sporns PB et al. *JAMA Network Open*.

Thi Dan Linh Nguyen-Kim	Department of Radiology and Neuroradiology, Stadtspital Zürich, Zürich, Switzerland
René Chapot	Department of Neuroradiology, Alfried-Krupp-Krankenhaus, Essen, Germany
Umut Yilmaz	Department of Neuroradiology, Saarland University Hospital, Homburg, Germany
Furene Wang	Department of Paediatrics, Khoo Teck Puat-National University Children's Medical Institute, National University Hospital, Singapore
Muhammad Ubaid Hafeez	Department of Neurology, Baylor College of Medicine, Houston, TX, USA
Flavio Requejo	Department of Neuroradiology, Hospital de Pediatría J.P. Garrahan, Buenos Aires, Argentina
Nicola Limbucci	Department of Interventional Neuroradiology, Careggi University Hospital, Florence, Italy
Birgit Kauffmann	Department of Pediatrics and Adolescent Medicine, Eltern-Kind-Zentrum Prof Hess, Klinikum Bremen Mitte, Bremen, Germany
Markus Möhlenbruch	Department of Neuroradiology, Heidelberg University Hospital, Heidelberg, Germany
Omid Nikoubashman	Department of Neuroradiology, Aachen University, Aachen, Germany
Peter D Schellinger	Department of Neurology and Neurogeriatrics, University Clinic of the Ruhr-Universität Bochum, Minden, Germany
Patricia Musolino	Department of Neurology, Massachusetts General Hospital, Harvard Medical School, Boston, MA, USA

© 2025 Sporns PB et al. *JAMA Network Open*.

Ali Alawieh	Department of Neurosurgery, Emory University School of Medicine, Atlanta, GA, USA
Jenny Wilson	Division of Pediatric Neurology, Oregon Health & Science University, Portland, OR, USA
Dominik Grieb	Department of Radiology and Neuroradiology, Sana Kliniken Duisburg, Duisburg, Germany; Department of Diagnostic and Interventional Neuroradiology, Medical School Hannover, Hannover, Germany
Alexandra S Gersing	Department of Neuroradiology, LMU University Hospital, LMU Munich, Munich, Germany
Thomas Liebig	Department of Neuroradiology, LMU University Hospital, LMU Munich, Munich, Germany
Martin Olivieri	Pediatric Thrombosis and Hemostasis Unit, Dr von Hauner Children's Hospital, LMU Munich, Munich, Germany

© 2025 Sporns PB et al. *JAMA Network Open*.
