# Supplementary material for: Intravenous Thrombolysis for Pediatric Acute Ischemic Stroke
Source: JAMA Netw Open. 2025 Oct 15;8(10):e2538191. doi: 10.1001/jamanetworkopen.2025.38191 (PMC12529207; doi:10.1001/jamanetworkopen.2025.38191)
Supplement: Supplement 2. — The SaveChildS Pro Investigators [file jamanetwopen-e2538191-s002.pdf]

Supplemental Online Content: Nonauthor Collaborators
*First name, last name, and suffix (if applicable) are required and will appear in PubMed.

*Group Name(s): Save ChildS Pro Investigators							
***First Name and Middle Initial(s)**	***Last Name**	***Suffix (eg, Jr, III)**	Academic Degrees	Institution	Location (city, state/province, country)	Role or Contribution, eg, chair, principal investigator	Group (if more than 1 Group listed in the byline) and/or Subgroup (eg, Steering Committee)
Peter B	Sporns						
Kartik	Bhatia						
Todd	Abruzzo						
Lisa	Pabst						
Stuart	Fraser						
Melissa G	Chung						
Warren	Lo						
Ahmed	Othman						
Sebastian	Steinmetz						
Ulf	Jensen-Kondering						
Stefan	Schob						
Daniel P O	Kaiser						
Wolfgang	Marik						
Christina	Wendl						
Ilka	Kleffner						
Hans	Henkes						
Hermann	Kraehling						
Thi Dan Linh	Nguyen-Kim						
René	Chapot						
Umut	Yilmaz						
Furene	Wang						
Muhammad Ubaid	Hafeez						
Flavio	Requejo						
Nicola	Limbucci						
Birgit	Kauffmann						
Markus	Möhlenbruch						
Omid	Nikoubashman						
Peter D	Schellinger						
Patricia	Musolino						

Supplemental Online Content: Nonauthor Collaborators
*First name, last name, and suffix (if applicable) are required and will appear in PubMed.

***First Name and Middle Initial(s)**	***Last Name**	***Suffix (eg, Jr, III)**	Academic Degrees	Institution	Location (city, state/province, country)	Role or Contribution, eg, chair, principal investigator	Group (if more than 1 Group listed in the byline) and/or Subgroup (eg, Steering Committee)
Ali	Alawieh						
Jenny	Wilson						
Dominik	Grieb						
Alexandra S	Gersing						
Thomas	Liebig						
Martin	Olivieri						
