# Supplementary material for: Intravenous Thrombolysis for Pediatric Acute Ischemic Stroke
Source: JAMA Netw Open. 2025 Oct 15;8(10):e2538191. doi: 10.1001/jamanetworkopen.2025.38191 (PMC12529207; doi:10.1001/jamanetworkopen.2025.38191)
Supplement: Supplement 3. — Data Sharing Statement [file jamanetwopen-e2538191-s003.pdf]

Data Sharing Statement

Sporns. Intravenous Thrombolysis for Pediatric Acute Ischemic Stroke. *JAMA Netw Open*.
Published October 15, 2025. doi:10.1001/jamanetworkopen.2025.38191

Data
Data available: No
